# Adverse Childhood Experiences and Problematic Social Media Use: Longitudinal Evidence Among Chinese Adolescents

**DOI:** 10.1002/jad.70053

**Published:** 2025-09-17

**Authors:** Qijia Cong, Mitch van Geel, Renate S. M. Buisman, Paul Vedder

**Affiliations:** ^1^ Institute of Education and Child Studies Leiden University Leiden the Netherlands

**Keywords:** adolescents, adverse childhood experiences, cumulative risk models, multiple informants, problematic social media use

## Abstract

**Introduction:**

Adverse childhood experiences (ACEs) are prevalent and have been linked to problematic social media use (PSMU) in adolescents. However, few prior studies focused on the longitudinal association and the functional nature between ACEs and PSMU. Drawing on the Compensatory Internet Use Theory and the Cumulative Risk Hypothesis, this study aimed to examine the relation between ACEs and PSMU as well as the cumulative effects of ACEs on PSMU using a three‐wave longitudinal design with multiple informant assessments of adolescent PSMU.

**Methods:**

A total of 264 Chinese adolescents (50.0% female; *M*_age_ = 13.91 years, SD = 0.76) and 234 parents (75.0% female; *M*_age(206)_ = 41.00 years, SD = 3.65) participated in the baseline measurement. Two separate sets of generalized linear mixed models (GLMMs) were performed to test the effects of ACEs on adolescent‐reported and parent‐reported PSMU.

**Results:**

Results from the GLMM analyses revealed that (1) exposure to ACEs significantly predicted adolescent‐reported PSMU (*b* = 0.17, *p* < 0.01), but not parent‐reported PSMU (*b* = 0.03, *p* = 0.65), and (2) the functional relation between cumulative ACEs and PSMU followed a linear pattern, irrespective of whether PSMU was reported by adolescents or parents. These findings provided empirical support for the Cumulative Risk Hypothesis, specifically aligning with the additive (linear) model.

**Conclusions:**

Earlier ACE exposure predicts subsequent adolescent PSMU; the functional relation between cumulative ACEs and PSMU is linear. This underscores the importance of addressing each ACE in prevention and intervention efforts aimed at mitigating adolescent PSMU.

## 1 Introduction

Adverse childhood experiences (ACEs) refer to a broad set of childhood intensive and frequently occurring stress experiences, including abuse, neglect, household dysfunction as well as peer, community, and collective violence (World Health Organization 2018). For decades, an array of studies have associated these early life traumatic experiences with subsequent maladaptive mental and behavioral problems, such as depression and anxiety (Samaey et al. 2023), bullying perpetration (Gómez‐Ortiz et al. 2016), and substance abuse (Damodaran et al. 2023). More recent research also suggests that experiencing ACEs could be a predictor for later problematic online behaviors like cyberbullying (Kircaburun et al. 2021), online gaming addiction (Grajewski and Dragan 2020), and problematic social media use (PSMU; Chegeni et al. 2023; Wilke et al. 2020). Even though several studies have explored the relation between ACEs and PSMU (Wang et al. 2022; Wilke et al. 2020; Worsley et al. 2018), nearly all have relied on cross‐sectional designs and exclusively on self‐reports. This approach presents significant limitations, including difficulties in establishing the temporal order of the relation between ACEs and PSMU (Camerini et al. 2020), and potential biases in self‐reported data (Austermann et al. 2021). In addition, although ample studies focus on the cumulative effects of ACEs on subsequent problem behaviors (Appleyard et al. 2005; Mersky et al. 2013; Yazgan et al. 2021), the functional relation between cumulative ACEs and adolescent PSMU remains underexplored and needs further examination. In our effort to address these shortcomings, the current study examined the longitudinal association between ACEs and PSMU as well as the functional nature of this relation in Chinese adolescents, using a multi‐informant (self and parent) assessment for adolescent PSMU.

### 1.1 ACEs and PSMU

With the advancement of internet‐ and mobile‐based technologies, social media has emerged as one of the most widely used recreational activities among youth worldwide (Gentzler et al. 2023; Lin et al. 2023). Social media usage is highly prevalent in China, with a considerable proportion of users being adolescents. Junior middle school students, in particular, engage with social media at rates more than 15 percentage points above the national average for underage netizens (China Internet Network Information Center 2023). The widespread accessibility of internet devices facilitates adolescents' susceptibility to excessively or constantly using social media, leading to PSMU (Bányai et al. 2017). PSMU is characterized by an uncontrollable engagement in social media, investing excessive time and effort into it, and leading to impairments in individuals' social activities, interpersonal relationships, health, and well‐being (Andreassen and Pallesen 2014; Sun and Zhang 2021). Most importantly, PSMU can adversely impact adolescents' functioning and development, with PSMU being associated with negative mental and behavioral health outcomes, including depression (Ergün et al. 2023; Li et al. 2018), anxiety (Barry et al. 2017), poor sleep quality (Lin et al. 2023), and even nonsuicidal self‐injury (Shen et al. 2024).

Given the high global prevalence of adolescent PSMU (12.2%; Meng et al. 2022) and its associated adverse health outcomes, researchers have increasingly focused on identifying its antecedents as possible anchors for prevention or interventions. Empirical research exploring the link between ACEs and PSMU is often grounded in the Compensatory Internet Use Theory (Kardefelt‐Winther 2014). This theory posits that negative life experiences, such as ACEs, may drive adolescents to turn to online activities as a means of alleviating negative emotions and fulfilling frustrated psychological needs. Social media offers a unique blend of real‐life and virtual interactions, allowing adolescents not only to escape their real‐life problems but also to connect with individuals or communities that provide a sense of belonging and social support (Yang et al. 2025). These online interactions through social media may help adolescents seek affirmation and emotional support, compensating for needs that may be unfulfilled in their offline lives (Wilke et al. 2020). This reliance on social media, however, could lead to prolonged and potentially problematic levels of engagement. In line with this theoretical framework, cross‐sectional associations between ACE exposure and problematic media use in youth have been found across both Western (Jackson et al. 2021; Morello et al. 2023) and Chinese contexts (Wang et al. 2022; Xue et al. 2025). These mostly cross‐sectional studies, however, neither allow for conclusions about the temporal order of changes in variables nor about their directionality (Camerini et al. 2020). For this type of information, longitudinal designs are needed, as they can offer greater insight into the developmental trajectories of the relation between ACEs and adolescent PSMU. However, longitudinal studies have been relatively scarce in this field (Xu et al. 2024). This underscores the critical need for further research to explore the long‐term associations between ACEs and PSMU.

### 1.2 Cumulative ACEs and PSMU

**Figure 1:**
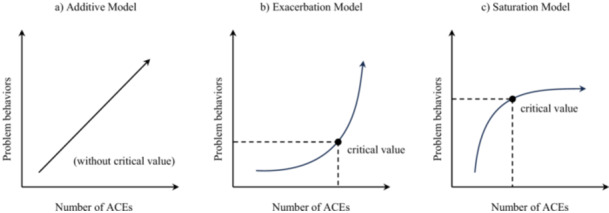
The functional relation models between cumulative ACEs and problem behaviors (figure designed by Rauer et al. 2008). (a) Additive Model. (b) Exacerbation Model. (c) Saturation Model.

ACEs rarely operate in isolation but frequently co‐occur (Turney 2020). The Cumulative Risk Hypothesis states that individuals' developmental outcomes are better predicted by the accumulation of risk factors rather than single‐specific risks (Rutter 1979). Within this approach, each predictor is assumed to carry equal weight, and cumulative risk models focus on the quantity rather than the intensity or pattern of risk exposure (Evans et al. 2013). Previous studies have explored the functional models of the risk‐outcome relation between cumulative ACEs and problem behaviors, with a particular focus on whether it adheres to a linear or nonlinear pattern. The *linear (additive) model* (see Figure 1a) posits that problematic behavioral outcomes increase steadily by one unit with each additional occurrence of ACEs, showing a “gradient effect” (Appleyard et al. 2005; Sameroff 2000). In contrast, the two other nonlinear models reveal potentially disproportionate associations between cumulative ACEs and problem behaviors. In the *exacerbation model* (see Figure 1b), exposure to childhood risks beyond the critical value can lead to a dramatic increase in problem behaviors, creating a “mass accumulation” effect (Rutter 1979). While the *saturation model* (see Figure 1c) suggests that after a specific number of cumulative ACEs, adding new ACEs could hardly influence the level of problem behaviors, presenting a leveling‐off effect (Gerard and Buehler 2004; Rauer et al. 2008).

Existing studies have examined the functional relations between cumulative risks and various behavioral outcomes. Evidence has been found for a linear relation between cumulative ACEs and aggressive behavior (Cui et al. 2013), but also for a nonlinear relation (exacerbation model) between cumulative childhood risks and disruptive behavior (Ashworth and Humphrey 2020), as well as for a nonlinear‐saturation model between cumulative ecological risks and smartphone addiction (Tian et al. 2023). These different findings highlight the need for further examination of functional relations between ACEs and PSMU, which is also important because different models may yield different practical implications. Specifically, a linear pattern suggests that each reduction in ACEs is critical and requires comprehensive interventions for PSMU (Appleyard et al. 2005). In an exacerbation model, the PSMU level accelerates rapidly once the number of ACEs reaches a critical point, leading to severe PSMU and heightened intervention difficulties. However, in this case, targeted interventions for individuals at the highest risk may be beneficial (Oldfield et al. 2015). The saturation model suggests that beyond a certain value of ACEs, the impact of each additional new ACE on PSMU decreases. This implies that the most effective interventions for PSMU should be achieved before reaching this critical value (Rauer et al. 2008).

### 1.3 Adolescent‐ and Parent‐Reports

The existing literature on adolescent PSMU has primarily relied on self‐reports (see, e.g., Acar et al. 2022; Bányai et al. 2017; Wang et al. 2022), even though self‐reports may be biased in several ways. For instance, self‐reports on problem behaviors may be confounded by social desirability and symptom denial (Austermann et al. 2021), which may lead adolescents to underreport their social media usage. In addition, relations between ACEs and adolescent behavior problems may be inflated due to shared method variance when all information is self‐reported (Lewis et al. 2010). To address these limitations, a multi‐informant approach, such as incorporating both parent and adolescent reports, can help reduce the measurement errors typical of single‐informant studies (Jepsen et al. 2012). As parents are often the first to notice their children's problem behaviors (Miller et al. 2017), their perspectives on adolescent media use are a valid source of information, providing a more comprehensive and balanced assessment of adolescent PSMU (Barry et al. 2017). Nevertheless, few studies included parent reports to evaluate adolescent PSMU, and the current study endeavors to address this gap in the literature.

### 1.4 The Present Study

PSMU is notably prevalent among Chinese middle school students, with rates reported ranging from 7.9% to 34.2% (Chao et al. 2023; Li et al. 2018). Early adolescence is a phase marked by an increased need for peer connection and identity exploration, which leads adolescents to prolonged engagement in social media, hoping to seek and find peer acceptance and validation (Nesi et al. 2017; Yang et al. 2021). Due to the high prevalence of PSMU among Chinese early adolescents and its potentially harmful effects on their development, the present study focuses on this age group and aims to examine the longitudinal association between cumulative ACEs and adolescent PSMU. Specifically, we tested whether exposure to ACEs could predict adolescent PSMU using a three‐wave longitudinal design, and subsequently identified the functional nature (linear, exacerbation, or saturation) of the longitudinal relation between cumulative ACEs and PSMU. Based on previous studies (Jackson et al. 2021; Wilke et al. 2020), we hypothesized that experiencing ACEs positively predicts adolescent PSMU (H1). Although several studies have identified different functional associations between cumulative risks and various problem behaviors, we based our second hypothesis on a Chinese longitudinal study relevant to our topic, which examined the link between cumulative risks and smartphone addiction (Tian et al. 2023), identifying a saturation model. Following this study, we hypothesized that a saturation model would best fit the relation between cumulative ACEs and PSMU (H2). Moreover, adolescents and parents hold different perspectives, which may both be valid to some extent (Buisman et al. 2020; Gromann et al. 2011). Therefore, H1 and H2 are tested in two separate longitudinal model sets with either adolescent‐report PSMU (adolescent model) or parent‐report PSMU (parent model) to discern potential differences in the longitudinal relation between cumulative ACEs and PSMU when using different reports of PSMU.

## 2 Methods

### 2.1 Participants

Participants were Chinese junior middle school students, together with their parents, in Qiqihar City, northeast China. Data collection was conducted in three waves during 1 year with half‐year intervals: April 2022, October 2022, and April 2023. Six classes from the 7th and 8th grades were randomly selected within the school, and 276 students and their main caregiver (the most involved parent in adolescents' study and life) were invited to participate voluntarily in our study. During the first wave of data collection (W1), a total of 264 adolescents (response rate 95.7%; 50.0% female; *M*_age_ = 13.91, SD = 0.76, range = 12–16) and 234 parents (response rate 84.9%; 75.0% female; *M*_age(206)_ = 41.00, SD = 3.65, range = 33–55) were included in the baseline measurement. Among the participants, 81.8% of adolescents were the only child in their family. Additionally, 39.6% of parents held a junior college degree or higher, and 44.3% of households reported an annual income exceeding 100,000 yuan (about 14,000 dollars). All adolescents and parents who completed the baseline assessment in Wave 1 were invited to participate in Wave 2 and Wave 3. At W2, 256 adolescents and 205 parents participated in the data collection. At W3, 243 adolescents and 216 parents completed the questionnaires.

### 2.2 Measures

#### 2.2.1 ACEs

ACEs were assessed using the Adverse Childhood Experiences ‐ International Questionnaire (ACE‐IQ) developed by the WHO (World Health Organization 2018). This 29‐item questionnaire contains 13 categories of childhood adversities: emotional neglect (2 items, P1–P2); physical neglect (3 items, P3–P5); emotional abuse (2 items, A1–A2); physical abuse (2 items, A3–A4); sexual abuse (4 items, A5–A8); living with substance abuser (1 item, F1); household mental health problems (1 item, F2); incarcerated household member (1 item, F3); parental death or separation/divorce (2 items, F4–F5); domestic violence (3 items, F6–F8); bullying (1 item, V1); witnessed community violence (3 items, V4–V6); and collective violence (4 items, V7–V10). In this study, adolescents were required to assess the ACEs they had undergone during their lives up to the day of data collection. Response options for each item were either dichotomous (0 = *no*, 1 = *yes*; Items F1–F5), based on a 5‐point Likert scale ranging from *Never* to *Always* (Items P1–P2), or based on a 4‐point Likert scale ranging from *Never* to *Many Times* (the rest of the items). The original ACE‐IQ offers two scoring algorithms. The *binary method* uses the lowest threshold for identifying ACEs, marking any occurrence of adversity as exposure (e.g., being yelled or screamed at *once* is identified as an affirmative response for emotional abuse). The *frequency method*, however, considers the degree of exposure, with thresholds that vary depending on the type of adversity (e.g., exposure to sexual abuse only requires being touched sexually *once*, whereas exposure to emotional abuse requires being yelled or screamed at *many times*). The original English version of ACE‐IQ and the guidance for analyzing ACE‐IQ can be accessed at https://cdn.who.int/media/docs/default-source/documents/child-maltreatment/ace-iq-guidance-for-analysing.pdf.

The Chinese version of the ACE‐IQ, revised and validated by Ho et al. (2019), has satisfactory reliability and validity in Chinese youth samples. Following the approach of Ho et al. (2019), we adopted the frequency method for estimating adolescent ACE exposure, which aligns with international standards and is suited for capturing the ACE profiles within Chinese samples (Wang et al. 2024; Xue et al. 2025). The ACE‐IQ assesses exposure across 13 categories of ACEs, allowing for a cumulative score that reflects the total number of different ACE categories the participant has experienced. Irrespective of the number of items per category, an affirmative response to at least one of the items signified exposure to that adversity (e.g., exposure to emotional abuse required at least one *Many Times* response from the two items in the “emotional abuse” category) and was counted as 1 point. Ultimately, the points from each category were summed to create a cumulative ACE score ranging from 0 to 13. The higher the score, the more ACEs the participant had experienced. Because the items in ACE‐IQ measured different domains of childhood adversities and these adversities may have little in common (Camille et al. 2023), using test–retest reliability instead of a measure of internal consistency was deemed preferable for estimating the reliability of the questionnaire. In this study, test–retest correlations of the cumulative ACE score between Wave 1 and Wave 2, Wave 2 and Wave 3, and Wave 1 and Wave 3 were found to be 0.74, 0.73, and 0.71, respectively.

#### 2.2.2 PSMU

PSMU was measured with the Social Media Disorder Scale (SMD scale; van den Eijnden et al. 2016). Participants assessed their social media usage during the past year on this 9‐item scale (e.g., “During the past year, have you regularly found that you can't think of anything else but the moment that you will be able to use social media again?”) with a binary scoring method (0 = *no*, 1 = *yes*). The total score ranged from 0 to 9, with higher scores indicating higher problematic social media usage levels during the past year. Fung (2019) validated the Chinese version of the SMD scale, and the scale showed good internal consistency (*α* = 0.75). In the present study, adolescents completed the Chinese version of the SMD scale to assess their own social media usage. Cronbach's alphas were 0.76, 0.83, and 0.84 for the three waves, respectively. Given that currently no Chinese parent‐report measures for PSMU are available, we rephrased the questions within the SMD scale to render them suitable for parental responses. For instance, the original question, “During the past year, have you often felt bad when you could not use social media?” was adapted for parental use to read, “During the past year, has your child often felt bad when he/she could not use social media?”. A similar method was employed in the study of Vadlin et al. (2015). Parents were required to estimate their children's PSMU at all three waves in the current study, and the Cronbach's alphas of the parental scale were 0.89, 0.90, and 0.90, respectively.

#### 2.2.3 Covariates

Existing review articles have found that gender, age, only child status, and family socioeconomic status (SES) were associated with PSMU (Li et al. 2014; Liang et al. 2024). Therefore, these variables were controlled as covariates in the main analyses. Adolescents and parents reported their gender and age in years. In addition, adolescents were asked whether they were the only child in their family. Family SES was measured using both parents' highest education and annual household income. In line with the study of Yang et al. (2022), parents' educational qualifications were divided into five levels: primary school and below, junior high school, ordinary or vocational high school, junior college, undergraduate and above. Annual household income was divided into four levels (Jin et al. 2017): 50,000 yuan and less (about 7000 dollars and less), between 50,000 and 100,000 yuan (between about 7000 and 14,000 dollars), between 100,000 and 200,000 yuan (between about 14,000 and 28,000 dollars), and more than 200,000 yuan. Considering that parents' education and household income were measured using different scales, these scores were standardized and subsequently summed to create a total score as an indicator of family SES, with higher scores representing a higher level of family SES.

### 2.3 Procedure

This study has obtained approval from the Institutional Ethics Review Board at Leiden University (reference number: ECPW‐2022/344). Background information letters about the study and informed consent were handed out to the adolescents, parents, and teachers before the baseline data collection. Adolescents and parents were informed that their participation was voluntary and that they could withdraw at any time during the study. After that, we invited each adolescent and the most involved parent who agreed on the informed consent to participate in Wave 1 data collection. Each adolescent–parent pair had a unique family code for three waves so that we could match their data when the whole data collection was finished. Both adolescents and parents got a reminder of their family code at the beginning of each wave. Adolescents were instructed to complete online questionnaires on Qualtrics independently in a separate and quiet room at school. Questionnaires were completed during self‐study hours to prevent students from missing any classes. Class teachers sent questionnaire links to each student's parent. During all three waves, parents completed online questionnaires at home. If parents forgot to complete the questionnaires, the class teachers would remind them 1 day later. After each wave, parents received a message of appreciation, and adolescents received stationery (bookmarks, notebooks, and highlighter pens) as a reward.

### 2.4 Statistical Analysis

#### 2.4.1 Missing Data

In the current study, missing data were 4.97%, 10.40%, and 12.29% in Wave 1, Wave 2, and Wave 3, respectively, for all the variables included in our models. We performed a missing value analysis. Little's MCAR test (Little 1988) was not significant (*χ* ^2^(2829) = 2920.93, *p* = 0.11), showing that the data were missing completely at random. Additionally, we compared the participants who were present in all three waves (*N*_adolescents_ = 243; *N*_parents_ = 158) to those who only participated in one wave (*N*_adolescents_ = 8; *N*_parents_ = 15) or two waves (*N*_adolescents_ = 13; *N*_parents_ = 83) on the variables included in subsequent models. Independent *t* tests (for adolescents' and parents' age, SES), *χ* ^2^ tests (for adolescents' and parents' gender, only child), and Mann–Whitney *U* tests (for ACEs, adolescent‐ and parent‐reported PSMU) were performed. Results showed that adolescents who participated in all three waves were significantly older than those who only participated in one wave (*t* = 4.63, *p* < 0.01, *d* = 0.91). Furthermore, the ACE scores of adolescents participating in all three waves were significantly lower than those of students participating in two waves (*Z* = −2.50, *p* < 0.05). In other comparisons for the remaining variables, we found no significant differences between participants who did and those who did not drop out between waves.

We used multiple imputation (MI) in R Version 4.3.0 (R Core Team 2023) to deal with the missing data, as MI is robust to various missing data mechanisms, including missing completely at random (MCAR) and missing at random (MAR; van Ginkel et al. 2020). In MI, missing values are estimated multiple times, generating several complete versions of the incomplete data set. Each of these data sets is subsequently analyzed using the intended statistical procedure, and the outcomes are amalgamated through specific combination procedures that consider the variation of the imputed values in standard errors and *p* values. MI has the advantage of retaining all available information, while simultaneously incorporating the uncertainty associated with the missing data into the statistical analysis (van Ginkel et al. 2020). We performed single‐level MI in the *mice* package by using predictive mean matching (PMM) as an imputation method (van Buuren and Groothuis‐Oudshoorn 2011). Missing data were imputed 50 times, with 100 iterations for each imputation. Autocorrelation function (ACF) plots revealed that all imputations tended to converge. All the variables (ACEs, adolescent‐ and parent‐reported PSMU, and covariates) were imputed in the models of the main analysis. The descriptives and correlations between variables were approximately the same in the imputed data sets compared with the non‐imputed data set (see Table 1). Therefore, we used the multiple imputed data sets for the main analyses.

**Table 1:** Descriptive statistics and correlations of the main variables.

	Mdn (ori)	IQR (ori)	1	2	3	4	5	6	7	8	9	10	11	12	13	14
Mdn (imp)			14.00	40.00	−0.37	0.01	0.01	0.00	0.00	0.00	2.00	2.00	2.00	1.00	1.00	1.00
IQR (imp)			1.00	5.00	3.41	3.66	3.40	1.00	1.00	1.00	3.00	3.00	4.00	4.00	4.00	4.00
1. W1 AR‐Age	14.00	1.00	—	0.04	−0.17**	−0.08	−0.15*	0.14*	0.08	0.05	0.18**	0.10	0.11	0.13*	0.01	0.04
2. W1 PR‐Age	41.00	4.25	0.06	—	0.12	0.09	0.10	−0.09	−0.09	−0.03	0.05	0.03	0.04	0.00	−0.02	0.08
3. W1 SES	−0.37	3.42	−0.17**	0.12	—	0.76***	0.74***	−0.09	−0.02	0.03	0.00	0.02	0.08	0.04	0.05	0.01
4. W2 SES	0.01	3.66	−0.08	0.10	0.78***	—	0.77***	−0.03	0.01	0.01	−0.03	−0.08	−0.04	0.06	0.00	0.01
5. W3 SES	0.01	3.40	−0.15*	0.10	0.79***	0.83***	—	−0.07	−0.05	−0.04	−0.06	−0.03	−0.01	0.04	0.01	0.01
6. W1 ACEs	0.00	1.00	0.14*	−0.06	−0.09	−0.03	−0.07	—	0.60***	0.56***	0.34***	0.30***	0.18**	0.32***	0.21**	0.24***
7. W2 ACEs	0.00	1.00	0.08	−0.10	−0.02	0.01	−0.04	0.62***	—	0.57***	0.33***	0.34***	0.22***	0.28***	0.27***	0.20**
8. W3 ACEs	0.00	1.00	0.05	−0.03	0.03	0.02	−0.04	0.63***	0.62***	—	0.30***	0.33***	0.30***	0.21**	0.23***	0.23***
9. W1 AR‐PSMU	2.00	3.00	0.18**	0.07	0.00	−0.04	−0.05	0.34***	0.35***	0.33***	—	0.56***	0.43***	0.34***	0.27***	0.19**
10. W2 AR‐PSMU	2.00	3.75	0.10	0.04	0.02	−0.08	−0.02	0.31***	0.35***	0.35***	0.58***	—	0.53***	0.25***	0.22***	0.21**
11. W3 AR‐PSMU	2.00	4.00	0.12	0.03	0.07	−0.04	−0.01	0.20**	0.24***	0.30***	0.47***	0.58***	—	0.21**	0.20**	0.21**
12. W1 PR‐PSMU	1.00	4.00	0.14*	0.00	0.04	0.07	0.06	0.32***	0.28***	0.24***	0.36***	0.27***	0.25***	—	0.41***	0.28***
13. W2 PR‐PSMU	1.00	4.00	−0.02	−0.03	0.07	−0.03	0.01	0.21**	0.27***	0.26***	0.32***	0.23**	0.25***	0.54***	—	0.34***
14. W3 PR‐PSMU	1.00	4.00	0.06	0.12	0.01	0.01	0.03	0.25***	0.20**	0.23**	0.20**	0.22**	0.22**	0.34***	0.50***	—

*Note:* Under the diagonal are correlations for original data; above the diagonal are correlations for imputed data.

Abbreviations: AR, adolescent‐reported; IQR (imp), interquartile range for imputed data; IQR (ori), interquartile range for original data; Mdn (imp), median for imputed data; Mdn (ori), median for original data; PR, parent‐reported; SES, family socioeconomic status; SES scores, standardized scores; W1, Wave 1; W2, Wave 2; W3, Wave 3.

* *p* < 0.05

** *p* < 0.01

*** *p* < 0.001.

#### 2.4.2 Analysis

Distributions of the predictive and outcome variables were inspected. The skewness and kurtosis statistics of ACEs (ACE_skewness_ = 27.49, ACE_kurtosis_ = 48.51) and the skewness of adolescent‐ and parent‐reported PSMU (AR‐PSMU_skewness_ = 11.55; PR‐PSMU_skewness_ = 11.18) were beyond the acceptable range (i.e., skewness < | 3.0 | and kurtosis < | 10.0 | ; Kline 2016), indicating that the data were not normally distributed. In view of the non‐normally distributed data, the Spearman rank correlations were used. Thereafter, we adopted two sets of generalized linear mixed models (GLMMs), which are a powerful tool for analyzing nonnormal data that involve random effects (Bolker et al. 2009), to separately test the effects of ACEs on adolescent‐ and parent‐reported PSMU. GLMM analysis was conducted in R using data in long format. The *mice* and *mitml* packages were employed to perform MI, fit GLMMs across imputed data sets, and pool the results (Grund et al. 2016; van Buuren and Groothuis‐Oudshoorn 2011). To examine the predictive effect of ACEs on adolescent PSMU, both lagged and concurrent ACE scores were included as predictors in the models. Specifically, the lagged ACE score was used to capture the temporal effect of ACE exposure from the previous wave, while the concurrent ACE score was included as a control to isolate the unique contribution of earlier ACE exposure. Continuous predictor variables were mean‐centered to allow easier interpretation of intercept and slope parameters (Peugh 2010). Adolescent‐reported and parent‐reported PSMU scores were specified as outcome variables in the adolescent and parent models, respectively. Using the pooled data, we first estimated a series of progressively complex unconditional multilevel models to determine the extent and structure of variance in PSMU. Specifically, we tested the unconditional means model (Model 1) to calculate the intra‐class correlation (ICC) and to assess the variance in PSMU within and between participants. Then we introduced time (waves) as a predictor to control for time‐related trends and tested the unconditional growth model with random intercepts only (Model 2), and the unconditional growth model with both random intercepts and slopes (Model 3). Including a random slope model in this study was theoretically and statistically justified, as three measurement points reached the minimum requirement for identifying random slopes in longitudinal models (Singer and Willett 2003; Snijders and Bosker 2012). This approach allowed us to capture whether adolescents differed not only in their baseline PSMU levels, but also in how their PSMU changes over time. Following these, the covariates (i.e., gender, age, “only child” status, and SES) were added as predictive variables in Model 4. In Model 5, nonsignificant covariates were removed, and the concurrent and the lagged ACE scores (the linear terms) were entered as predictors to examine whether experiencing early adversities supplied extra variance over the covariates in predicting adolescent‐ and parent‐reported PSMU. In Model 6, the squared concurrent and the squared lagged ACE scores were added as quadratic terms besides the linear terms to test the functional form of the relation between cumulative ACEs and PSMU. The effects of the quadratic term could be used to discern the functional relation natures in variable models (Cohen et al. 2003). If the coefficient of the quadratic ACE term is significantly positive, the relation between cumulative ACEs and PSMU aligns with the exacerbation model. Conversely, a significantly negative coefficient suggests conformity with the saturation model. In the case of a nonsignificant coefficient of the squared ACE term, it implies that the nature of the relation is just linear. The likelihood ratio test (LRT) was used to evaluate whether the model fit improved (Grund et al. 2016).

## 3 Results

Table 1 presents the descriptive statistics and correlations of the main variables before and after the imputation. The range of ACE scores was 0–9 in Wave 1, 0–8 in Wave 2, and 0–7 in Wave 3. In Wave 1, 52.3% of adolescents reported no ACEs, 26.9% reported experiencing one ACE, 12.1% reported two, and 8.7% reported three or more. The scores for PSMU, as reported by both adolescents and parents, ranged from 0 to 9 across three waves. The correlation analysis indicated that W1 adolescent age was positively associated with ACEs, adolescent‐reported PSMU, and parent‐reported PSMU at W1. Besides, ACEs, adolescent‐reported PSMU, and parent‐reported PSMU were positively associated with each other across three waves. The results of the Wilcoxon signed rank test showed that there were no significant differences between adolescent‐ and parent‐reported PSMU (*Z* = −0.39, *p* = 0.70).

### 3.1 Adolescent Model With Adolescent‐Reported PSMU

The results of the GLMMs on adolescent‐reported PSMU are presented in Table 2. Based on the unconditional means model (Model 1), we calculated the ICC, which was 0.63, meaning that approximately two‐thirds of the total variation in PSMU lies between adolescents. This suggests that larger differences exist among adolescents in their average PSMU scores compared with the differences in PSMU scores within an adolescent over time, implying a need for multilevel models. Model 2 showed no increasing or decreasing trend in PSMU over time, suggesting there was no effect of time. The LRT indicated that Model 2 did not fit the data better than Model 1 (*F*(1, 6770.59) = 2.76, *p* = 0.10). In Model 3, we tested whether adolescent PSMU differed in both intercepts and slopes. The results showed that the model had a large eigenvalue and was nearly unidentifiable, which means that the model fit of Model 3 was too complex for the data, and there were no random slopes in the change of adolescent PSMU. Therefore, we continued with Model 2 with only the random intercepts. Model 4 included adolescent age, gender, only child status, and SES as predictors. Adolescent gender was kept in the model because of a marginally significant effect (*b* = −0.28, *p* = 0.056), and the other nonsignificant covariates were removed from the subsequent models. Model 4 did not fit the data better than Model 2 (*F*(4, 2570.82) = 1.58, *p* = 0.18). In Model 5, we entered the linear terms of the concurrent and lagged ACE scores to test H1, which led to a significant improvement compared with Model 2 (*F*(3, 2517.38) = 244.27, *p* < 0.001). Both concurrent and lagged ACE scores were significant predictors of adolescent PSMU. Specifically, the lagged ACE score (*b* = 0.17, *p* < 0.01) was positively associated with subsequent PSMU, suggesting that more early ACE exposure predicted higher levels of subsequent PSMU, which supported our first hypothesis. Adolescent gender positively predicted PSMU (*b* = −0.33, *p* < 0.05), with girls tending to have higher levels of PSMU than boys. Model 6 added the squared concurrent and lagged ACE scores as quadratic terms based on Model 5 to further examine the second hypothesis. The results revealed that in addition to the significant predictions of gender and the linear terms, the coefficients of the squared concurrent ACE score (*b* = −0.01, *p* = 0.57) and the squared lagged ACE score (*b* = −0.01, *p* = 0.93) were both negative but not statistically significant. This indicates that the relation between cumulative ACEs and adolescent‐reported PSMU was linear, which refuted H2. In addition, Model 6 did not fit the data significantly better than Model 5 (*F*(2, 1053.29) = −0.12, *p* = 1.00).

**Table 2:** Results of the pooled GLMMs for adolescent‐reported PSMU.

Parameters	Model 1		Model 2		Model 3		Model 4		Model 5		Model 6	
	Coefficient (SE)	*t*	Coefficient (SE)	*t*	Coefficient (SE)	*t*	Coefficient (SE)	*t*	Coefficient (SE)	*t*	Coefficient (SE)	*t*
Intercept	0.37 (0.07)	5.26***	0.37 (0.07)	5.30***	0.34 (0.07)	5.04***	0.46 (0.17)	2.70**	0.49 (0.11)	4.58***	0.51 (0.11)	4.75***
Wave			0.05 (0.03)	1.71	−0.01 (0.05)	−0.18	0.02 (0.04)	0.40	0.08 (0.06)	1.22	0.07 (0.06)	1.21
AR‐Age							0.07 (0.06)	1.19				
AR‐Gender							−0.28 (0.15)	−1.91	−0.33 (0.15)	−2.26*	−0.33 (0.14)	−2.39*
Only Child							0.07 (0.16)	0.40				
SES							−0.03 (0.02)	−1.21				
Concurrent ACEs									0.29 (0.05)	6.27***	0.31 (0.06)	5.02***
Lagged ACEs									0.17 (0.06)	2.89**	0.16 (0.06)	2.82**
Concurrent ACEs squared											−0.01 (0.02)	−0.56
Lagged ACEs squared											−0.01 (0.07)	−0.10
*σ* ^2^_intercept_	0.91		0.91		0.82		0.85		0.82		0.80	
*σ* ^2^_slope_	—		—		0.08		—		—		—	
Cov_(intercept, slope)_	—		—		0.01		—		—		—	

*Note:* Gender (0 = *female*; 1 = *male*), Only Child = Whether the only child in family (0 = *no*; 1 = *yes*)

Abbreviation: AR, adolescent‐reported.

* *p* < 0.05

** *p* < 0.01

*** *p* < 0.001.

### 3.2 Parent Model With Parent‐Reported PSMU

The results of the GLMMs on parent‐reported PSMU are displayed in Table 3. Based on Model 1, the ICC was estimated at 0.69, which suggests that 69% of the total variance in parent‐reported PSMU resides between parents, indicating more differences in parent‐reported PSMU scores between parents than within a parent across time. Model 2 showed that there was no increase or decrease in PSMU over time, and Model 2 did not fit the data better than Model 1 (*F*(1, 335.45) = 0.05, *p* = 0.83). In Model 3, we tested whether parent‐reported PSMU differed in the intercepts and slopes. There was a significant improvement when comparing Model 3 to Model 2 (*F*(2, 5706.58) = 55.58, *p* < 0.001), indicating that parent‐reported PSMU differed in the change rate over time. Based on Model 3 with random slopes, parent age, gender, only child status and SES were added in Model 4. The significant parent gender was kept, and the other three covariates were nonsignificant and removed from the subsequent models. Model 4 did not fit the data better than Model 3 (*F*(4, 622.94) = 1.32, *p* = 0.26). In Model 5, we added the linear terms, and Model 5 showed a good model fit and led to a significant improvement compared with Model 3 (*F*(3, 2824.02) = 296.80, *p* < 0.001). Concurrent ACE exposure was a significant predictor of parent‐reported PSMU (*b* = 0.30, *p* < 0.001), with higher ACE scores of adolescents related to higher levels of PSMU from parents' perspective. However, the lagged ACE score did not significantly predict PSMU (*b* = 0.06, *p* = 0.62), suggesting that earlier ACEs adolescents experienced have limited long‐term effects on subsequent parent‐reported PSMU and not supporting H1. Parent gender positively predicted PSMU (*b* = 0.44, *p* < 0.05), with fathers perceiving their children to have higher levels of PSMU than mothers. In Model 6, the squared concurrent and lagged ACE scores were entered next to the linear terms to test H2. The results showed that, except for the significant prediction of parent gender and the concurrent ACE score, the coefficients of the squared concurrent ACE score (*b* = −0.05, *p* = 0.09) and the squared lagged ACE score (*b* = −0.03, *p* = 0.80) were not significant. This suggests that the relation between cumulative ACEs and parent‐reported PSMU was also linear. Hypothesis 2 was rejected. Additionally, Model 6 did not fit the data significantly better than Model 5 (*F*(2, 1352.48) = 1.46, *p* = 0.23). Table 4 shows model fit indices and comparison results of all models in the adolescent and the parent models.

**Table 3:** Results of the pooled GLMMs for parent‐reported PSMU.

Parameters	Model 1		Model 2		Model 3		Model 4		Model 5		Model 6	
	Coefficient (SE)	*t*	Coefficient (SE)	*t*	Coefficient (SE)	*t*	Coefficient (SE)	*t*	Coefficient (SE)	*t*	Coefficient (SE)	*t*
Intercept	0.32 (0.09)	3.69***	0.32 (0.09)	3.69***	0.19 (0.09)	2.09*	0.09 (0.20)	0.46	0.08 (0.14)	0.53	0.17 (0.15)	1.16
Wave			−0.01 (0.04)	−0.23	0.001 (0.08)	0.02	0.004 (0.08)	0.05	−0.13 (0.17)	−0.77	−0.17 (0.17)	−0.96
PR‐Age							−0.01 (0.03)	−0.19				
PR‐Gender							0.42 (0.19)	2.20*	0.44 (0.21)	2.05*	0.43 (0.21)	2.02*
Only Child							0.04 (0.20)	0.19				
SES							−0.02 (0.03)	−0.57				
Concurrent ACEs									0.30 (0.07)	4.22***	0.44 (0.11)	4.20***
Lagged ACEs									0.06 (0.12)	0.49	0.03 (0.13)	0.27
Concurrent ACEs squared											−0.05 (0.03)	−1.71
Lagged ACEs squared											−0.03 (0.10)	−0.25
*σ* ^2^_intercept_	1.24		1.24		1.69		1.68		4.90		4.81	
*σ* ^2^_slope_	—		—		0.37		0.37		1.70		1.71	
Cov_(intercept, slope)_	—		—		−0.39		−0.39		−2.49		−2.48	

*Note:* Gender (0 = *female*; 1 = *male*), Only Child = Whether the only child in family (0 = *no*; 1 = *yes*)

Abbreviation: PR, parent‐reported.

* *p* < 0.05

** *p* < 0.01

*** *p* < 0.001.

**Table 4:** Fit indices and model comparison.

		Model fit indices				Model comparison	
	Models	AIC	BIC	Log‐Likelihood	Models	*F*	*p*
Adolescent model	Model 1	2968.82	2978.17	−1482.41			
	Model 2	2967.71	2981.74	−1480.86	M2 vs. M1	2.76	0.10
	Model 3	—	—	—	M3 vs. M2	—	—
	Model 4	2964.12	3006.19	−1473.06	M4 vs. M2	1.58	0.18
	Model 5	2006.47	2032.09	−997.24	M5 vs. M2	244.27	0.00
	Model 6	2009.96	2044.11	−996.98	M6 vs. M5	−0.12	1.00
Parent model	Model 1	3380.90	3390.25	−1688.45			
	Model 2	3382.23	3396.26	−1688.12	M2 vs. M1	0.05	0.83
	Model 3	3258.01	3281.39	−1624.01	M3 vs. M2	55.58	0.00
	Model 4	3373.06	3405.78	−1679.53	M4 vs. M3	1.32	0.26
	Model 5	2109.55	2143.71	−1046.78	M5 vs. M3	296.80	0.00
	Model 6	2108.86	2151.55	−1044.43	M6 vs. M5	1.46	0.23

## 4 Discussion

The present study contributed to the understanding of the longitudinal association between ACEs and PSMU in Chinese adolescents. Results indicated that experiencing ACEs was a significant predictor of adolescent‐reported PSMU. We did not find the lagged effects of ACEs on parent‐reported PSMU, though the concurrent associations were significant across time points. In addition, the functional relation between cumulative ACEs and PSMU was found to fit a linear model in both the adolescent model and the parent model. These findings challenged the limitations of prior cross‐sectional studies and highlighted the long‐term impact of ACEs on PSMU development during adolescence. Meanwhile, the findings offered practical insights for improving the prevention and interventions aimed at addressing PSMU.

The results of this study indicated that a greater number of ACEs predicted higher PSMU levels in adolescents. ACEs essentially mirror adverse environments and a deficiency of support from family, school, and society, which are crucial for adolescents' health and well‐being (Kim et al. 2021). Therefore, adolescents with ACEs may experience unmet relationship needs due to a lack of support (Worsley et al. 2018). In line with the compensatory internet use theory (Kardefelt‐Winther 2014), to compensate for the resulting frustration or stress in real life, these adolescents may turn to online platforms. These platforms could provide an escape from or alleviate the pain from their frustrating experiences. This context may mitigate negative feelings and signal a desire for social support (Wilke et al. 2020). The underlying desire to seek solace and support online may encourage adolescents to spend more time on social media, potentially leading to subsequent problematic use patterns. However, this finding was only supported in the adolescent model and not in the parent model. This discrepancy may reflect a divergence between adolescent and parent perceptions of maladjustment behaviors (De Los Reyes and Kazdin 2005; Vierhaus et al. 2016). Subjective psychological experiences, such as unmet emotional or relational needs resulting from ACEs, may be difficult for parents to detect, whereas adolescents may be more directly aware of their own internal states. Furthermore, prior studies have shown that parents tend to misjudge the frequency and emotional impact of their children's social media use, particularly when parent–child communication is limited (George and Odgers 2015; Livingstone and Helsper 2008). It is also possible that in families with higher exposure to ACEs, parents' awareness of adolescent behaviors may be further compromised due to strained familial relationships or reduced effective parenting (Van Heel et al. 2019). This may explain why, although ACE exposure was concurrently associated with parent‐reported PSMU, no long‐term effects across time were observed.

Contrary to the second hypothesis, findings from both the adolescent model and the parent model indicated that the functional relation between cumulative ACEs and PSMU is linear and consistent with the additive model (see Figure 1a). Specifically, each ACE adolescent experienced correlated with a similar step‐wise increase in PSMU levels, while there did not appear to be a particular threshold value for ACEs beyond which the development of PSMU either accelerates or decelerates. Though unexpected, previous longitudinal evidence has suggested linear associations between cumulative childhood risks and later problem behaviors in adolescents (Appleyard et al. 2005; Wong et al. 2013). Speculatively, this pattern may reflect that ACEs, as significant threats in adolescents' daily lives, and as measured in the current study, are profoundly related to their social functioning and development in deleterious ways. Existing research has pointed out the strong negative effects of emotional neglect (Buisman et al. 2018), physical and sexual abuse (Meyerson et al. 2002) may have on child development. These particular ACEs were all included as single items in our ACE measure. A concentration on such significant risks may lend support to a linear model without “safe thresholds,” as each increase in ACEs could consistently translate to a corresponding rise in adverse outcomes (Gerard and Buehler 2004; Raviv et al. 2010). In addition, this finding may be linked to our focus on PSMU. In contrast to externalizing behaviors, social media use is readily accessible and generally hardly discouraged (Bányai et al. 2017). Thus, adolescents may not feel compelled to restrain their social media use, at least compared with externalizing behaviors that may evoke a more negative response from their environment. As a result, when faced with increasing numbers of ACEs, adolescents may continue to increase their PSMU levels.

It is noteworthy that the random slopes model provided a better fit for the parent‐reported PSMU, whereas the adolescent‐reported PSMU was better represented with the random intercepts only model. One potential explanation for this divergence in model structure may lie in the variability of parental perceptions across time. Specifically, the inclusion of a random slope for time in the parent model suggests that parents differ not only in their baseline perceptions of adolescent PSMU but also in the trajectories of these perceptions over time. Such temporal variability could be influenced by random factors such as the quality of parental monitoring and parent–child communication (Beyens et al. 2024; Wallace 2021), particularly in families with ACEs. In contrast, adolescent self‐reports may more consistently reflect internal experiences of PSMU, less influenced by shifting external interpretations. This structural distinction may also partly account for why ACEs significantly predicted adolescent‐reported PSMU rather than parent‐reported PSMU: adolescents themselves may be more directly attuned to the psychological mechanisms linking early adversities and problematic media use, whereas parental observations are potentially filtered through varying degrees of awareness or concern, which shift over time. These findings underscore the value of incorporating multi‐informant approaches and flexible modeling strategies when examining complex behavioral outcomes in developmental settings.

The findings of this study provide valuable insights for future prevention and interventions targeted at adolescents facing challenges related to excessive social media use. Adolescents with ACEs may be reluctant to disclose their traumatic experiences (Arseneault et al. 2010). When, for example, clinicians or school counselors notice excessive social media use among adolescents, they could explore whether these individuals have previously experienced or are currently exposed to ACEs. Although our research did not establish a causal relation between ACEs and PSMU, our findings suggest that it may be worthwhile to explore whether helping adolescents deal with ACEs could contribute to diminishing their PSMU (Barboza 2018). Additionally, our results suggest that adolescents with higher ACE scores may face adversities across multiple domains, such as family, school as well as community, and they tend to exhibit higher levels of PSMU than their peers with low ACE scores. Though causal relations still have to be established in future research, as suggested by other researchers (Oldfield et al. 2015; Raviv et al. 2010), it may be worthwhile to explore if adolescents with ACEs across different domains need more comprehensive intervention approaches, whereas those with fewer ACEs could respond well to more moderate, targeted support such as individual or group‐based programs.

Several limitations of the present study should be acknowledged. First, we did not examine the associations between different types of ACEs and PSMU as our sample size did not allow for robust analysis of specific ACE categories. Future studies with larger samples could consider investigating specific ACE domains separately to uncover potential differences in how various types of ACEs relate to adolescent PSMU. Second, all measures used in this study were retrospective, which may render ACEs and PSMU reports vulnerable to recall bias (Nagata et al. 2023). Future work could utilize prospective designs to further enhance our understanding of the association between ACEs and PSMU. Third, the ACE‐IQ we used in this study could not distinguish between ACEs that happened a long time ago and recent ACEs. Without this distinction, it is impossible to determine whether the observed relations between ACEs and PSMU are attributable to the long‐term impact of past ACEs or the more immediate impacts of recent adverse experiences. Future research could benefit from addressing this distinction to better understand the immediate and long‐term effects of ACEs on adolescent PSMU. Finally, the sample of this study was selected from one northeastern city in China, which may result in sample homogeneity to some extent. Therefore, caution should be exercised when attempting to generalize our findings to other regions of China (Jiang et al. 2022). Given that our study represents the initial attempts to investigate the longitudinal relation between ACEs and PSMU, future research endeavors could involve nationwide sampling and broaden the sample scope to validate and analyze the generalizability of the findings from our study.

## 5 Conclusion

The current study employed a multi‐informant approach to examine the longitudinal association between ACEs and PSMU within a Chinese adolescent sample. The results indicated that exposure to ACEs positively predicts PSMU levels in adolescents. Notably, the functional relation between cumulative ACEs and PSMU was found to be linear when PSMU was reported by adolescents as well as when reported by parents. These findings provide empirical support for the additive model of cumulative ACEs, suggesting that each additional ACE adolescent experienced links to a corresponding increase in their PSMU levels. The findings of this study provide important implications for future practical work, emphasizing the importance of interventions targeted at adolescent PSMU by proactively identifying and mitigating the negative impacts of ACEs.

## Author Contributions

**Qijia Cong:** conceptualization, data curation, formal analysis, investigation, writing – original draft, writing – review and editing. **Mitch van Geel:** conceptualization, methodology, project administration, writing – review and editing. **Renate S. M. Buisman:** formal analysis, methodology, writing – review and editing. **Paul Vedder:** conceptualization, writing – review and editing, supervision.

## Conflicts of Interest

The authors declare no conflicts of interest.

## Data Availability

Data will be made available on appropriate request.
